# Anthropogenic Pollution Intervenes the Recovery Processes of Soil Archaeal Community Composition and Diversity From Flooding

**DOI:** 10.3389/fmicb.2019.02285

**Published:** 2019-10-02

**Authors:** Yu Wang, Yiguo Hong, Maohua Ma, Shengjun Wu, Huub J. M. Op den Camp, Guibing Zhu, Wei Zhang, Fei Ye

**Affiliations:** ^1^Institute of Environmental Research at Greater Bay Area, Key Laboratory for Water Quality and Conservation of the Pearl River Delta, Ministry of Education, Guangzhou University, Guangzhou, China; ^2^Chongqing Institute of Green and Intelligent Technology, Chinese Academy of Sciences, Chongqing, China; ^3^Department of Microbiology, Institute for Water and Wetland Research, Radboud University Nijmegen, Nijmegen, Netherlands; ^4^Key Laboratory of Drinking Water Science and Technology, Research Center for Eco-Environmental Sciences, Chinese Academy of Sciences, Beijing, China

**Keywords:** archaea, autogenic recovery, disturbance, human impact, riparian zone, three gorges reservoir

## Abstract

Archaea play vital roles in global biogeochemical cycles, particularly in nitrification and methanogenesis. The recovery of archaeal community following disturbance is essential for maintaining the stability of ecosystem function. To examine whether the archaeal community could recover from water flooding and assess the influence of anthropogenic pollution on the autogenic recovery, soil samples from two riparian zones with contrasting pollution background were investigated. Collected samples in each area were divided into three groups of reference, flooding, and recovery according to the flooded state of each site. The results showed that the archaeal abundance was resilient to the disturbances of both water flooding and anthropogenic pollution. More similar community composition and diversity appeared between the recovery and reference groups in the area with low anthropogenic pollution. It indicated that high anthropogenic pollution could result in less resilience of archaeal community. The co-occurrence network further revealed that the archaeal community in the area of low anthropogenic pollution exhibited more associations suggesting a higher ecosystem stability. The better recovery of archaeal community was associated with the high resilience ability. The *Nitrososphaerales* was the key taxon maintaining the better recovery of the archaeal community from the disturbances due to its high resilience index and quantitative dominance. Overall, archaeal community has the capability of autogenic recovery, the process of which might be intervened by anthropogenic pollution, and then potentially affects the ecosystem functions of the riparian system.

## Introduction

Microbial community composition is critical for predicting rates of ecosystem processes, which is often ignored comparing with plant (Allison and Martiny, 2008). In fact, microbial communities respond more rapidly than plant communities to changing environmental conditions (Harris, 2009), and play central roles in ecosystem processes in many ways, such as driving the Earth’s biogeochemical cycles (Griffiths and Philippot, 2013), affecting plant productivity and community dynamics (Wardle et al., 2004; van der Heijden et al., 2008), and re-establishing function and microbial diversity in ecosystem restoration (Harris, 2009). Recovery after being disturbed is essential for microbe, since most natural environments are subject to disturbances over time. The measurements of soil microbial community structure and composition are increasingly being applied to assess the response of ecosystems to disturbances and as an indicator of ecosystem recovery (Harris, 2003; van Dijk et al., 2009; Lewis et al., 2010). Detailing the response of microbial communities to disturbances and the recovery processes are important to understand the associations between microbial community structure and functional properties of ecosystems (Yannarell et al., 2007).

Disturbances in soil environment refer to the changes in physical or chemical condition (especially from anthropogenic sources) (Glasby and Underwood, 1996). In the context of global change, four types of disturbance have been frequently studied: increased CO_2_ concentrations, temperature changes, mineral fertilization (N/P/K), and enrichment with C substrates (Allison and Martiny, 2008). More than 80% of the mineral fertilization, temperature, and C amendment researches observed significant impacts of disturbances on microbial composition (Allison and Martiny, 2008). An effect of elevated CO_2_ concentrations was found as many as 60% of the studies (Allison and Martiny, 2008). In addition, the dry/wet cycles represent another common disturbance on soil microbial communities, which was predicted to grow more influential with future climate change (Bapiri et al., 2010). The dry/wet cycles occur in many occasions like irrigation in agricultural soil (Burger et al., 2005), precipitation in arid and semiarid ecosystems (Sponseller, 2007), and water fluctuation in riparian soil (Wang et al., 2016). One of the most well-known consequences is that the soil microbial biomass and its activity increase sharply after rewetting, and then a large amount of gaseous carbon and nitrogen erupt from the soil, named as “Birch effect” (Birch, 1958). Hence, the anthropogenic disturbance and dry/wet cycles are two major environmental disturbances regulating the soil microbial composition and dynamics.

Microbial communities respond to environmental disturbance differently, restructuring the microbial communities is the most common response (Vellend, 2010). In other cases, resistance and resilience are two patterns of microbial community response to disturbances: resistance means that microbial composition could remain unchanged after disturbance due to a high degree of metabolic flexibility or physiological tolerance (Meyer et al., 2004); while resilience means that even if microbial communities do change as a result of disturbance, the communities might quickly return to its composition before disturbance (Allison and Martiny, 2008). Previous studies investigated the influence of one individual disturbance (e.g., hurricane, drying-rewetting cycles, compaction, and fire) to soil microbial community (Yannarell et al., 2007; Bapiri et al., 2010; Hartmann et al., 2014; Lee et al., 2017). Actually, current threats to soil microbial community are usually a consequence of different sources of co-occurring disturbances that operate synergistically. When facing a joint effect of disturbances, how do the microbial community and recovery process respond are poorly understood.

Three Gorges Dam is the most important hydroelectric project along Yangtze River (Nilsson et al., 2005; Shi, 2011; Xu et al., 2011). The riparian zone of the three gorges reservoir (TGR) is directly impacted by the periodical fluctuation of water level, which is impounded to 175 m in winter and discharged to 145 m in summer in one consecutive inundation cycle (China Three Gorges Corporation, 2017). The frequent and dramatic water level fluctuation in the riparian zone modify the quantity of available nutrients (Ye et al., 2015), and reduce oxygen availability in the soil thereby controlling microbial community composition and function (Wu et al., 2015). As an aggravating factor, the direct discharge of industrial and domestic sewage without treatment also intensify the enrichment of nutrient substances in the riparian soils (Su and Zhang, 2010; Xian et al., 2013), and are acting synergistically with disturbance from water level fluctuation.

In this study, we targeted the archaeal community in the riparian zone of the TGR, and hypothesized that (i) the archaeal community had the ability to recover from the disturbances and (ii) the recovery process of the archaeal community might be affected by the joint effect of those two disturbances. To test these hypotheses this study (i) examined whether archaeal community has the ability of autogenic recovery in riparian zones; (ii) assessed the recovery of archaeal communities from flooding in riparian zones with contrasting anthropogenic pollution levels; and (iii) revealed the key taxa with properties of resistance and resilience in the recovery process. To achieve these, we specifically applied multi-step approaches to cover different potentially useful microbial indicators including the quantification of archaea by quantitative PCR (qPCR), the community description via amplicon sequencing analysis followed by the calculation of resilience and resistance indices, and the description of the community organization and potential interactions via network analysis.

## Materials and Methods

### Study Area and Sampling Strategy

**FIGURE 1:**
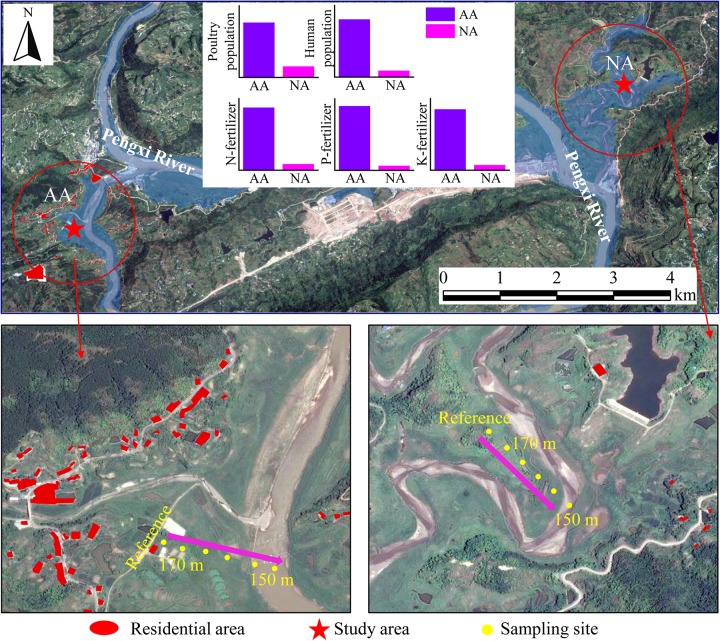
Location of the study areas (*red stars*). The residential areas around NA and AA within 1 km (*red circles*) are highlighted in *red*. The extent of the flooded riparian zone upon reaching the highest water level is shown in *blue background*. Along an elevation gradient of 150–170 m, sampling sites were set up at 5 m intervals. Samples taken at 175.5 m, soil not flooded, were used as reference for undisturbed soil. The bar charts indicate the different intensities of anthropogenic pollution in NA and AA represented by poultry population, human population, and fertilizing amount, the data of which are presented in Supplementary Table S2.

The soil in the riparian zone of this study is submerged for 39–273 days depending on elevation. While the water level starts to drop, the submerged soil re-exposes and returns to an unflooded state (Supplementary Table S1). Two riparian zones with entirely contrasting intensities of anthropogenic pollution (Figure 1 and Supplementary Table S2) were chosen: (i) Baijiaxi riparian zone (31°09′02″ N, 108°33′45″ E) and (ii) Xiaohe riparian zone (31°07′37″ N, 108°28′35″ E). The Baijiaxi area located in a wetland nature reserve, where the limited population pressure triggers much less anthropogenic pollution, is taken as a natural area (NA) in this study. The Xiaohe area is close to a village about 7.5 km upstream of Baijiaxi area. Due to the imperfect drainage system, large amounts of untreated sewage in Xiaohe are discharged into the adjacent river through the riparian zone. Furthermore, the nearby livestock and poultry breeding further intensify the pollutant input into Xiaohe area, which represents an anthropogenic area (AA) in this study. In addition, the two study areas are characterized by similar zonal soil type, soil texture, and meteorological conditions (Ye et al., 2019).

The water level in both the NA and AA fluctuates periodically between the highest level of 174–175 m and the lowest level of 150 m. To take advantage of the *in situ* flooding gradient, samples were taken at 5 m intervals in a transect along the elevational gradient. A total of four sampling surveys were conducted at October 10, 2013 and April 1, 2014, September 23, 2014, and May 6, 2015, representing the beginning and later stages of water flooding in two consecutive inundation cycles (Supplementary Figure S1).

Referred to previous study (Lee et al., 2017), 24 collected samples in both NA and AA were divided into three groups: samples collected from the unflooded sites of 175.5 m were set as reference group; and sites below the water level were set as flooding group; the sites which were flooded previously and were above the water level when sampling were set as recovery group (Supplementary Table S1). A total of 4 reference soils, 7 recovery soils, and 13 flooding affected soils were taken in each study area. Soil samples from 0 to 10 cm depth were collected using a stainless-steel core sampler and a Petersen grab for samples above and below water level, respectively. Bare surface soil samples were taken to minimize the impacts of plants and to maximize the comparability between samples. Subsamples were collected in triplicate at each sampling site, and then mixed thoroughly to form one composite sample. A portion of each composite soil sample was stored at 4°C for physicochemical analysis, and the remaining soil was stored at −20°C before nucleic acid extraction.

### Determination of Soil Physicochemical Properties

Soil pH was determined at a soil to milliQ water ratio of 1:5 using a pH analyzer. Soil moisture was measured using gravimetric method with 105°C oven-drying for 48 h. ${\text{NH}}_{4}^{+}$ and ${\text{NO}}_{3}^{-}$ were extracted from soil with 2 mol L^–1^ KCl at a soil to solution ratio of 1:5, and then measured using a flow injection analyzer (FIA Star 5000, FOSS Tecator, Sweden). The contents of total carbon (TC), total nitrogen (TN), total sulfur (TS), and the C/N ratio in soils were determined with an element analyzer (Vario EL cube, Elementar, Germany). Organic matter (OM) was analyzed through loss-on-ignition method at 550°C (Dean, 1974). Soil ferrous iron (Fe^2+^) was extracted with 0.1 mol L^–1^ Al_2_(SO_4_)_3_ solution at pH of 2.5, and the Fe^2+^ concentration in the supernatants was measured colorimetric using *o*-phenanthroline at 520 nm absorbance (UV-1750, Shimadzu, Japan). The Fe^3+^ concentration resulted from the difference between hydroxylamine hydrochloride reduced Fe^2+^ and Fe^2+^ measured directly [Bao, 2000 (In Chinese)]. Since the Fe^2+^–Fe^3+^ system often acts as an electron carrier, we employed the Fe^2+^/Fe^3+^ ratio as an indicator for estimating the redox environment in soil (Zheng et al., 2001). All analyses were performed in triplicate.

### DNA Extraction and Quantitative PCR

Approximately 0.25 g soil was used to extract DNA from each sample using the PowerSoil DNA Isolation Kit (Mobio, United States). The quantity and quality of extracted DNA were checked with a NanoVue Plus Spectrophotometer (GE Healthcare, United Kingdom). The abundance of the 16S rRNA gene of archaea was measured in triplicate on a Mastercycler (Eppendorf, Germany) with specific primer pairs A364aF–A934bR (Burggraf et al., 1997; Großkopf et al., 1998). Standard curve was built using a 10-fold serial dilution of plasmids containing the targeted fragments of archaeal 16S rRNA gene. Each qPCR reaction contained 10 μL SYBR^®^ Premix Ex Taq^TM^ II (Takara, Japan), 0.8 μL of each primer (10 pmol μL^–1^), 1 μL DMSO, and 1 μL of template. qPCR assay was as follow: 95°C for 2 min, followed by 40 cycles of 95°C for 20 s, 63°C for 60 s, and 68°C for 60 s (Ye et al., 2016). The qPCR efficiencies were 84.0-92.1% (*R*^2^ > 0.990).

### Sequencing and Processing of Archaeal 16S rRNA Genes

The V3–V5 region of the archaeal 16S rRNA gene was amplified by PCR using primers Arch344F (5′-barcode-ACGGGGYGCAGCAGGCGCGA-3′) and Arch915R (5′-GTGCTCCCCCGCCAATTCCT-3′) (Bernd et al., 2012). The PCR products were gel purified using the AxyPrep DNA Gel Extraction Kit (Axygen, United States). Subsequently, purified amplicons were pooled in equal amounts and paired-end sequenced on an Illumina MiSeq PE300 platform^1^. Samples at 160 m elevation were excluded from high-throughput sequencing analysis due to the very close flooding/exposure rotation with that of 165 m.

Raw sequence data were conducted using Trimmomatic (version 0.30). The merged sequences were quality-filtered, and sequences which could not meet the following parameters were discarded: sequence length <300 bp, average quality score ≥ 20 over a 50 bp sliding window, and no ambiguous bases. Operational taxonomic units (OTUs) were picked at 97% sequence similarity using the standard UPARSE pipeline within USEARCH (vsrsion 7.0^2^). Taxa were identified with SILVA rRNA database (Release 128^3^). The phylogenetic analysis of dominant taxa sequences (relative abundance > 1%) was conducted using FastTree (version 2.1.3) based on approximately maximum-likelihood. To compare archaea community diversity in different samples/groups on an equal basis, sequences (20,826-41,684 reads per sample) were rarefied to 20,826 reads per sample according to the sample with the lowest number of reads.

### Nucleotide Sequence Accession Number

The Illumina sequencing data were submitted to the NCBI Sequence Read Archive^4^ under accession No. PRJNA394057.

### Statistical Analysis

Alpha diversity (Shannon diversity, Chao1 richness, and Shannon-based species evenness) and rarefaction analysis were calculated using Mothur (version v.1.30.1) based on the rarefied sequence data. The differences in soil properties between study areas were conducted with Mann–Whitney *U*-test using SPSS Statistics 20.0 for Windows (IBM, United States). The differences of archaeal abundance, alpha diversity, and among groups were calculated based on multiple Kruskal–Wallis using SPSS Statistics 20.0 for Windows (IBM, United States). Analysis of similarity (ANOSIM; permutations = 999) was used to compare the archaeal community (OTU level) among groups based on Bray–Curtis distance using program R version 3.0.1 (vegan package). Redundancy analyses (RDAs) of archaeal community structures (OTU level) and correlations with soil properties were performed using the CANOCO 5 software based on log-transformed data. Graphs were generated using ORIGIN 9.0 software.

### Calculation of Resistance and Resilience

The resistance (RS) and resilience (RL) indices were employed to quantitatively assess the response of the archaeal community to disturbance. The RS index was calculated for archaea gene abundance and diversity, accounting for differences caused by a disturbance, using the following equation (Orwin and Wardle, 2004):

$$\text{RS}=1-\frac{2\,\left|C_{0}-P_{0}\right|}{\left(C_{0}+\left|C_{0}-P_{0}\right|\right)}$$

where *C*_0_ and *P*_0_ are the values of variables in the reference and flooding groups, respectively. The values of RS range between −1 and +1 with +1 representing no effect of disturbance (maximal resistance).

The RL index was calculated for the same variables after disturbance according to equation as (Orwin and Wardle, 2004):

$$\text{RL}\,\left(x\right)=\frac{2\,\left|C_{0}-P_{0}\right|}{\left(\left|C_{0}-P_{0}\right|+\left|C_{x}-P_{x}\right|\right)}\,-1$$

where *C*_0_ was equal to *C*_*x*_ in this study, which is the values of variables in the reference groups. *P*_0_ and *P*_*x*_ are the values of those variables in the flooding and recovery groups, respectively. The values of RL also range between −1 and +1 with +1 represents complete recovery (maximal resilience).

### Network Analysis

Network analysis was carried out to reveal the co-occurrence patterns of archaeal communities in different study areas. To visualize the associations in networks, correlation matrixes were constructed by calculating the possible pairwise Spearman’s rank correlations based on genus level (Faust and Raes, 2012). The Spearman’s rank correlation coefficient (ρ) absolute value > 0.6 and the *P*-value < 0.01 was considered as a statistically robust correlation between genera (taxa) (Hu et al., 2016). The nodes and edges in the networks represented archaeal taxa and strong correlations between nodes, respectively. Topological characteristics of the networks were used to describe the associations among archaeal taxa (Ma et al., 2016). The Spearman’s rank correlations were calculated with Python 2.6, and networks were visualized with Gephi (Bastian et al., 2009).

## Results

### Soil Properties in NA and AA

**FIGURE 2:**
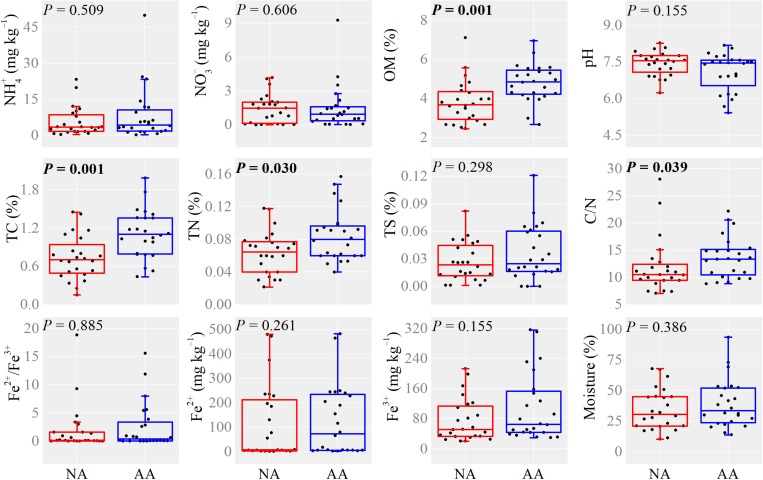
Boxplots of soil properties in NA and AA. Single dot represents the average value in each sampling elevation. *P*-values in bold indicate significant differences at 0.05 level based on Mann–Whitney *U*-test.

The higher anthropogenic impact in AA increased the content of OM, TC, TN, and C/N ratio significantly (Mann–Whitney *U*-test, *P* < 0.05, Figure 2). No significant difference on ${\text{NH}}_{4}^{+}$, ${\text{NO}}_{3}^{-}$, TS, Fe^2+^/Fe^3+^, Fe^2+^, Fe^3+^, and moisture was observed between the two areas (Figure 2). The soil ${\text{NO}}_{3}^{-}$, OM, TN, C/N, and Fe^3+^ in the dry (recovery) soils did not show significant difference with the flooding soils in both areas (Mann–Whitney *U*-test, *P* > 0.05, Supplementary Figure S2). Besides, water flooding significantly increased the soil TC, TS, Fe^2+^/Fe^3+^, and Fe^2+^ in AA (Mann–Whitney *U*-test, *P* < 0.05), whereas no significant differences were observed in NA (Supplementary Figure S2).

### Archaeal Abundance and Diversity in Different Sample Groups

**FIGURE 3:**
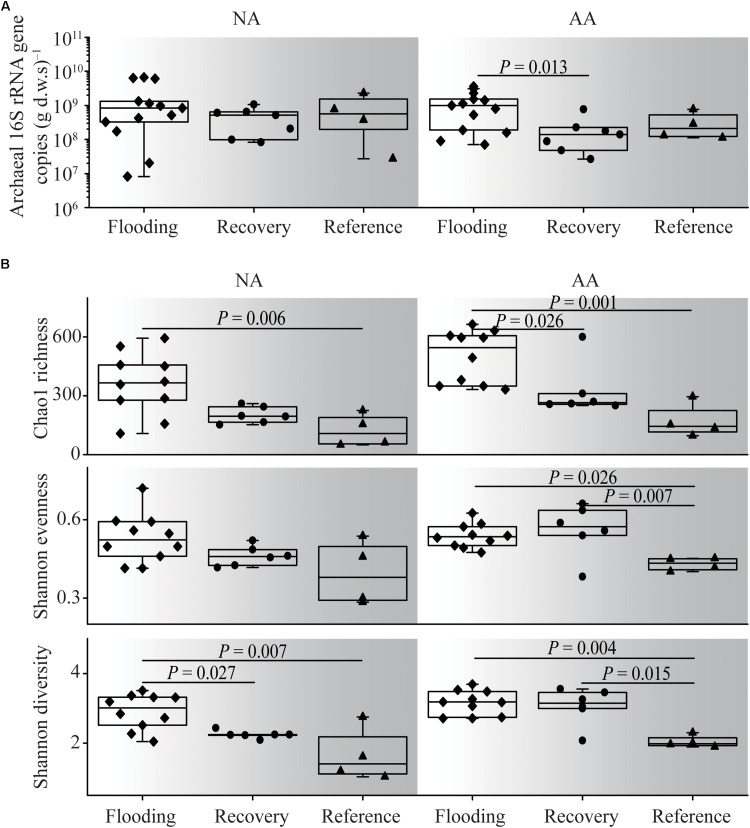
Archaeal abundance **(A)** and alpha diversity **(B)** of the flooding, recovery, and reference groups in NA and AA. Significant differences at *P* < 0.05 level based on multiple Kruskal–Wallis test were shown.

The average archaeal abundance in the NA flooding group was 1.9 × 10^9^ copies (g d.w.s., gram dry weight soil)^–1^, and no significant difference from the recovery and reference groups was observed (multiple Kruskal–Wallis test, *P* > 0.05, Figure 3A). By contrast in AA, the flooding group showed the highest abundance on average [1.2 × 10^9^ copies (g d.w.s.)^–1^], which was significantly higher than in the recovery group (multiple Kruskal–Wallis test, *P* = 0.013). In general, the average abundances in the recovery groups were close to those of the reference groups both in NA and AA [4.6 × 10^8^ vs. 8.6 × 10^8^ copies (g d.w.s.)^–1^ and 2.1 × 10^8^ vs. 3.2 × 10^8^ copies (g d.w.s.)^–1^].

Archaeal alpha diversity indices within the different flooding groups were generally higher than those of the reference groups in both NA and AA (Figure 3B). The Shannon diversity, Shannon evenness, and Chao1 richness in the recovery group were generally in between the flooding and reference groups; however, different patterns were observed in the two study areas. In NA, significant difference (multiple Kruskal–Wallis test, *P* = 0.027) was observed between the recovery and flooding groups on Shannon diversity, while no significant difference was observed between the recovery and reference group. Contrarily, in AA Shannon evenness and Shannon diversity in the recovery group differed significantly from those in the reference group (multiple Kruskal–Wallis test, *P* < 0.01). Therefore, the recovery soil in NA was more comparable to the reference soil with respect to alpha diversity.

### Archaeal Community Composition in NA and AA

**FIGURE 4:**
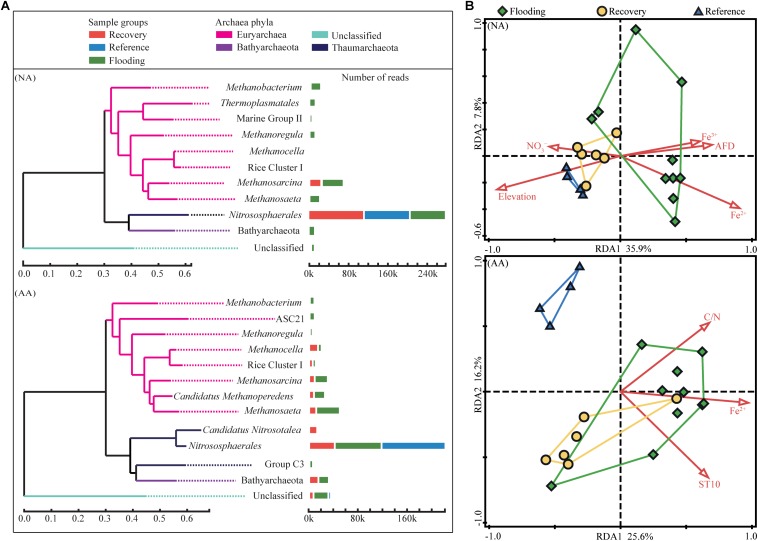
Relative abundance of dominant taxa (relative abundance > 1%) in the three sample groups **(A)**, and redundancy analysis of archaeal community structure in NA and AA **(B)**. Colors indicate the sample groups, i.e., flooding (*green*), recovery (*yellow*), or reference (*blue*). The statistically significant (*P* < 0.05) explanatory variables are shown with *red arrows*.

The most abundant taxa (primarily at the family/genus level; relative abundance >1%) across all NA sample groups accounted for 92.7% of all sequences (Figure 4A). Water level fluctuation and the resulting periodical exposure to air in particularly affected the proportion of ammonia-oxidizing archaea and methanogens. The relative abundance of ammonia-oxidizing archaea, which was the main component of Thaumarchaeota, increased from 38.9% in the flooding group to 75.8% in the recovery group and 95.7% in the reference group in NA (Supplementary Figure S3). In contrast, the euryarchaeotal methanogens decreased in the reference (3.1%) and recovery (20.8%) groups compared to the flooding group (47.6%, Supplementary Figure S3).

In AA, the most abundant taxa represented 95.7% of total sequences (Figure 4A). The Thaumarchaeota represented 37.8 and 47.6%, the Euryarchaeota represented 44.3 and 36.3%, and the Bathyarchaeota corresponded to 6.4 and 10.1% of the total sequences in flooding and recovery groups, respectively (Supplementary Figure S4). In the reference group, the proportions of those three phyla (Thaumarchaeota, Euryarchaeota, and Bathyarchaeota represented 93.6, 2.9, and 0%, respectively) were largely different from the flooding and recovery groups. The relative abundance of ammonia-oxidizing archaea in the reference group in AA was 93.6%, which decreased to 46.7% in the recovery group and 35.6% in the flooding groups (Supplementary Figure S3). Similarly to NA, the methanogens had the lowest relative abundance in the reference group, and increased to 30.5% in the recovery group and 34.6% in the flooding groups (Supplementary Figure S3).

Redundancy analysis was used to compare the community structure across the sample groups in NA and AA. The archaeal communities in the flooding group appeared to be less related to each other than the communities in the other two groups. The sampling sites in the two study areas were at least partly clustered according to the sample group (Figure 4B). This separation was in particular evident for NA, where the community structure was mostly governed by the sampling elevation (forward selection pseudo *F* = 8.60, *P* = 0.001) and the associated flooding duration (AFD, days) (forward selection pseudo *F* = 1.60, *P* = 0.045). In contrast, the flooding and recovery sites in AA grouped distantly from the reference sites.

Community compositions on OTU level were further compared between the three groups by an ANOSIM similarity test (Table 1). The community compositions of the flooding groups were significantly different from the reference groups both in NA (*R* = 0.504, *P* = 0.002) and AA (*R* = 0.340, *P* = 0.027). The recovery group in NA differed significantly (*R* = 0.307, *P* = 0.010) from the flooding group, while no significant difference was observed between the recovery and reference groups (*R* = 0.147, *P* = 0.154), which indicated more similar community compositions between the recovery and reference groups. In AA, the recovery group was significantly different from the flooding group (*R* = 0.228, *P* = 0.033) and reference group (*R* = 0.564, *P* = 0.011), and showed a stronger difference from the reference group according to the higher *R*-value.

**TABLE 1:** Comparison of community compositions among the three sample groups in NA and AA based on ANOSIM analysis.

**Study area**	**Comparison**	**ANOSIM^a^**	
		
		***R***	***P***
NA	Recovery vs. flooding	**0.307^b^**	**0.010**
	Recovery vs. reference	0.147	0.154
	Flooding vs. reference	**0.504**	**0.002**
AA	Recovery vs. flooding	**0.228**	**0.033**
	Recovery vs. reference	**0.564**	**0.011**
	Flooding vs. reference	**0.340**	**0.027**

*^*a*^ANOSIM was calculated on OTU level based on Bray–Curtis distance with 999 permutation implying the dissimilarity between two groups. ^*b*^Significant values are highlighted with bold.*

### Resilience and Resistance of Archaeal Community

**FIGURE 5:**
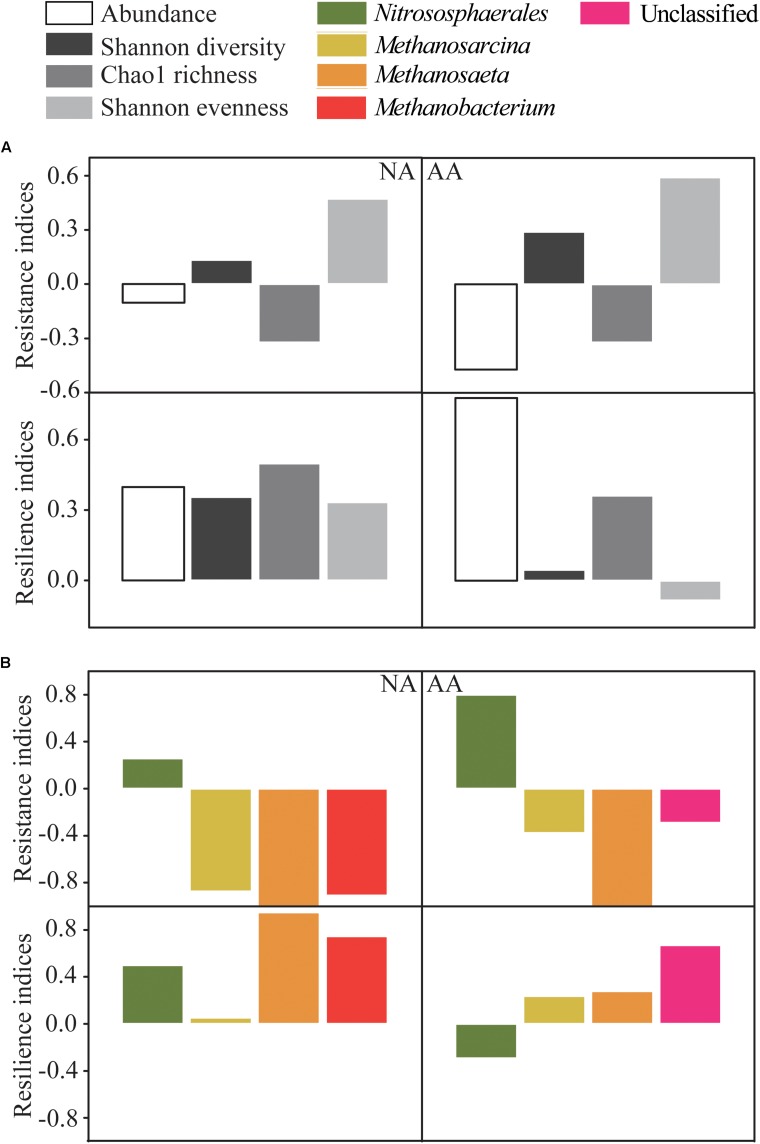
Resistance and resilience indices of abundance and alpha diversity of archaeal community **(A)**, and the four most abundant taxa at each sampling area **(B)**. The alpha diversity was calculated as Shannon diversity, Chao1 richness, and Shannon evenness.

The archaeal abundance showed low resistance as indicated by the negative RS index for NA and AA (−0.10 and −0.47, respectively) (Figure 5A). In contrast, the RL index was as high as 0.40 and 0.78, respectively, suggesting a higher ability of archaeal abundances to recover from flooding in both areas. All RS indices of alpha diversity in AA (0.29 of Shannon diversity, −0.32 of Chao1 richness, 0.59 of Shannon evenness) were slightly higher than those in NA (0.13 of Shannon diversity, −0.32 of Chao1 richness, 0.47 of Shannon evenness). However, the RL indices of alpha diversity in NA (0.33–0.50) were much higher than those in AA (−0.08–0.36), indicating a better recovery of the archaeal diversity in NA.

In an additional step, we compared the RS and RL indices of the four most abundant taxa which included 82.2 and 71.4% of the total sequences in NA and AA, respectively (Figure 5B). The highest positive RS values in both study areas were obtained for *Nitrososphaerales* (0.26 and 0.80 in NA and AA, respectively) as the most abundant taxon accounting for 60.9 and 48.1% of the sequences in NA and AA, respectively. In addition, the most abundant taxon *Nitrososphaerales* showed a high RL value (0.50) in NA, while it was negative in AA (−0.29). The other three tested taxa (*Methanobacterium*, *Methanosaeta*, and *Methanosarcina*) showed negative RS values and positive RL values whenever they occurred in the respective areas. Finally, sequences considered as unclassified showed negative RS value (−0.29), but rather high RL value (0.67) in AA.

### Co-occurrence Patterns of the Archaeal Communities in NA and AA

**FIGURE 6:**
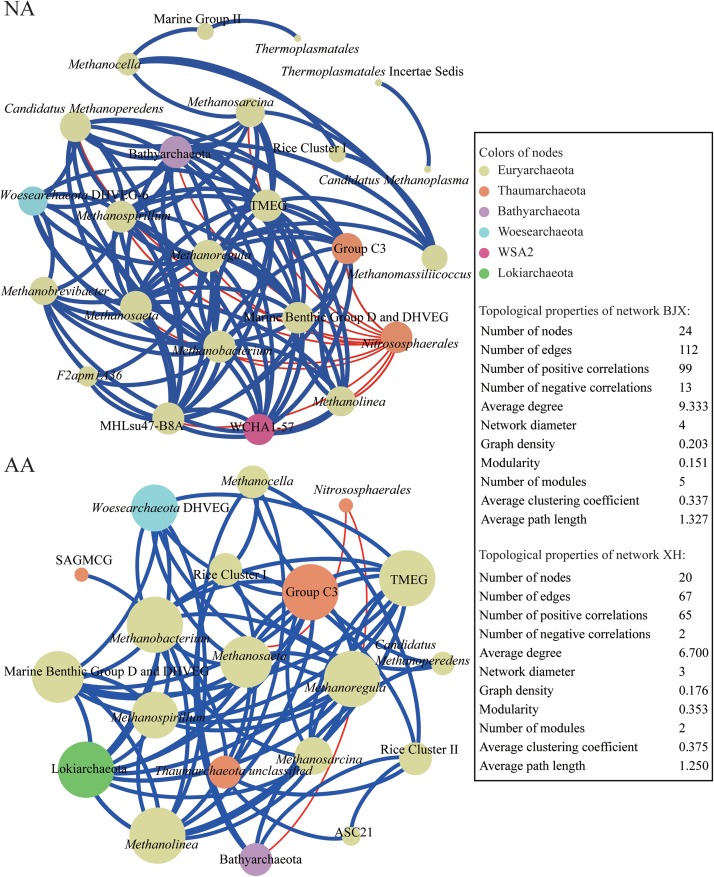
Co-occurring network of archaeal communities in NA and AA based on correlation analysis. The nodes in network are colored by phylum. The connections in the network indicate strong (ρ > 0.6) and significant (*P* < 0.01) correlations. The size of each node in proportion to the number of connections (i.e., degree); the thickness of each connection between two nodes (i.e., edge) in proportion to the Spearman’s correlation coefficient. A *blue edge* indicates a positive interaction between two individual nodes, while a *red edge* indicates a negative interaction.

We explored the co-occurrence patterns of the archaeal community in NA and AA using network analysis (Figure 6). The two networks showed comparable number of nodes with 24 and 20 in NA and AA, respectively. There were almost double as much edges were observed in the NA networks. Among these edges, 99 positive correlations and 13 negative correlations were observed in NA, while 65 positive and 2 negative correlations were observed in AA. Furthermore, the average degree (*avgK*, that is the average connectivity of nodes which suggested a more complex network with higher *avgK*) was greater in the NA network (9.333) than in the AA network (6.700). Other topological properties of the two networks, such as the average path length (that is the average network distance between all pairs of nodes), and the clustering coefficient (i.e., how and what degree nodes tend to cluster together) were very comparable. Finally, the modularity index (that is what extent a network could be naturally divided) was 0.151 vs. 0.353 in NA and AA. Overall, the co-occurrence networks in the two areas suggested stronger interactions of the archaeal community in NA than AA.

## Discussion

### Recovery Ability of Archaea Community

In this study, the recovery of the archaeal community was assessed by comparing the community structure in different disturbing stages which were flooding (disturbing), recovery, and reference. No significant impact of flooding on total archaeal abundance was observed. As a whole, the abundance of archaea has a strong ability to resist disturbance, which is probably due to, on the one hand, a high adaptation to diverse and stressful conditions of archaea, either by their low-permeability membranes or specific catabolic pathways (Valentine, 2007); on the other hand, high functional diversity of archaea, for instance, Euryarchaeota emerge in anaerobic conditions (Hu et al., 2013), and aerobic conditions increase the number of the Thaumarchaeota (Pester et al., 2011), eventually maintain the abundance at a stable level. It was coincident to a certain extent that the relative abundance of Euryarchaeota (52.0 and 44.2% in NA and AA, respectively) was highest in flooding group, whereas Thaumarchaeota (95.9 and 93.6% in NA and AA, respectively) was highest in reference group (Supplementary Figure S4). Moreover, the flooding groups in both NA and AA had the most abundant euryarchaeotal methanogens (47.6 and 34.6% in relative abundance), and the least ammonia-oxidizing archaea (38.9 and 35.6% in relative abundance) compared with the reference and recovery groups (Supplementary Figure S3). It can be expected that the flooding disturbance may increase methane generation but decrease ammonia oxidation.

In contrast, the community diversity, which is a parameter frequently associated with the ecosystem stability (Tilman et al., 2006; Wang and Loreau, 2016), appeared to be more sensitive to the disturbances. Although the flooding significantly increased the alpha diversity, the diversity in the recovery group showed a tendency to return to the predisturbance level (reference group) after the flooding receded. It was very obvious from the NA samples which showed comparable Shannon diversity level in the recovery and reference groups. Studies performed within coal mine have identified that microbial community with good recovery ability harbor comparable diversity level in both recovery and reference soils (Li et al., 2014; Quadros et al., 2016). Therefore, a better recovery of archaeal community in NA was supported by the relatively close values of diversity between the recovery and reference groups. Similar patterns were also observed in the community composition in NA, in which the recovery group was different from the flooding group but was relatively similar with the reference group (Table 1). The RDA plot further demonstrated that the community compositions in NA were clustered together by the sample groups. The distributions of archaeal community compositions were significantly impacted by the variables (${\text{NO}}_{2}^{-}$, Fe^2+^, and flooding duration) closely related to the elevation. By contrast, less variables significantly impacted the community composition in AA indicating more factors in addition to the measured variables also shaping the community composition.

### Anthropogenic Impact of Archaea Recovery

As two basic properties of ecological stability, resistance and resilience were pervasive in evaluating the response of microorganisms to disturbance (Thion and Prosser, 2014; Ng et al., 2015; Chen et al., 2017). By calculating resistance (RS) and resilience (RL) indices, we found that the RS index of archaeal abundance was negative in both sampling areas, hence, it can be concluded that the relative stability of archaeal abundance was mostly ascribed to the high resilience. Similarly, the better recovery of diversity in NA was also associated with its high resilience. The *Nitrososphaerales* was the dominant phylotype of archaea in a wide variety of environments (Bates et al., 2011) and represented almost half of the retrieved sequences in this study. As a key component, the recovery of the *Nitrososphaerales* was important for the entire archaeal community. Since the RS values were comparable in the two areas, the better recovery of the archaeal community composition in NA was probably ascribed to the higher RL values of the *Nitrososphaerales*. Therefore, it is speculated that the ammonia oxidation by archaea in the riparian zone might be substantial playing an important role in nitrogen removal in the TGR.

Co-occurrence networks are used to infer potential ecological associations between organisms and are useful tools to assess the ecosystem stability for a long time (May, 1972; Pimm, 1984; Montoya et al., 2006). The co-occurrence network in NA showed more complex associations. The number of edges (significant interactions, *P* < 0.01), average degree (*avgK*), average path length, and diameter of the network in NA were higher than those in AA. Previous studies proposed that the existence of species of a higher association with each another significantly increased both resistance and resilience against perturbation (May, 1972; Stouffer and Bascompte, 2011). This proposition was further identified in microbial ecology that the highly complex networks meant high resistance to disturbances and could be used to indicate ecosystem stability (Peura et al., 2015). Hence, the higher degree of species association and complex network in NA might indicate a more stable community. The diversity was a very common indicator to predict ecosystem stability (Tilman et al., 2006; Wang and Loreau, 2016). However, the two study areas, which had distinct recovery property, did not show obvious difference concerning diversity (Figure 3). Therefore, we could infer that the diversity cannot be a sign of ecosystem stability on its own. The association between species, as showed in the networks, would be of certain importance.

The recovery ability of archaea community in AA was weaker than in NA probably associated with the higher pollution level in AA. It has been reported that the resilience of methane oxidation activity was observed only at low pollution level, while microbial diversity was decreased and no recovery was observed at high level of pollution (Deng et al., 2011). Besides, a combined effect of disturbances may also reduce the recovery of microbial community. A previous study showed that respiration of soils contaminated with metals was very sensitive to subsequent heat or salt disturbances, which proves that disturbed systems have less energy to deal with additional disturbances (Tobor-Kapłon et al., 2006). Thus, microorganisms from highly polluted soils have lowered resources due to the energy consumption of detoxification and damage repair caused by the first disturbance, making it hard to deal with any additional disturbance (Calow, 1991; Kuperman and Carreiro, 1997). Therefore, the soil in NA exhibited a better recovery compared to that in AA where both flooding and anthropogenic pollution occurred. These results suggested that archaeal communities in the riparian soils have the ability of autogenic recovery from water flooding, whereas the autogenic recovery process would be intervened by human impact.

### Significance to Reservoir Ecosystem

The full operation of the TGR has resulted in periodic disturbances of water level fluctuation to the riparian zone. Currently, the original terrestrial ecosystem is at an early stage of secondary succession resulting in the extinction of local plant species and reduction of diversity (Bao et al., 2015). In the meantime, the microbial community, which is a key component of the riparian ecosystem, is also in a process of adaptation to the changed environmental conditions. It can be foreseen that this adaptation would eventually lead to a more stable community in response to the disturbance, which has been shown in other studies (Garcia-Villaraco Velasco et al., 2009; Stres et al., 2010). A stable and high resilience microbial community is of great significance in re-establishing ecosystem functioning during restoration (Harris, 2009), because of the role in regulating biogeochemical cycles (Griffiths and Philippot, 2013). However, there was usually a threshold intensity above which the microbial community could never recover and turn to alternative states (Gao et al., 2011; Griffiths and Philippot, 2013). The archaeal community in the riparian zone of the TGR has the ability to recover from flooding disturbance like in NA embedded in the context of low anthropogenic pollution, rather than in AA with a higher human impact. Moreover, high anthropogenic pollution levels may accelerate the methane production, whereas weaken the ammonia oxidation in the riparian zone. Therefore, in the recovery process, great efforts should be made to avoid human interference from outside scale, to facilitate the autogenic succession of the riparian ecosystem achieving high ecosystem stability.

## Data Availability Statement

The datasets generated for this study can be found in NCBI Sequence Read Archive, PRJNA394057.

## Author Contributions

YW, SW, GZ, and WZ designed the study. FY and MM performed the field work and laboratory work. YW, YH, and FY analyzed the data. YW and FY wrote the manuscript. HO contributed to the writing. All authors reviewed the manuscript.

## Conflict of Interest

The authors declare that the research was conducted in the absence of any commercial or financial relationships that could be construed as a potential conflict of interest.

## Footnotes

1 http://www.Majorbio.com

2 http://drive5.com/uparse/

3 http://www.arb-silva.de

4 http://www.ncbi.nlm.nih.gov/sra/
