# Supplementary material for: Anthropogenic Pollution Intervenes the Recovery Processes of Soil Archaeal Community Composition and Diversity From Flooding
Source: Front Microbiol. 2019 Oct 2;10:2285. doi: 10.3389/fmicb.2019.02285 (PMC6783558; doi:10.3389/fmicb.2019.02285)
Supplement: Supplementary file 1 [file Data_Sheet_1.PDF]

Supplementary Table 1 The continuous flooded or exposure duration (days) of different elevations by the sampling day in both areas.

Sampling date	Water level (m)	Water flooded (F) or exposed to air (E) when sampling	Flooding or exposure duration (days)					
			Reference (175.5 m)	170 m	165 m	160 m	155 m	150 m
2013.10.10	168.6	F	Flooding unaffected	-	21	28	37	60
		E		252	-	-	-	-
2014.4.1	161.3	F		-	-	200	209	232
		E		70	39	-	-	-
2014.9.23	168.5	F		-	9	22	26	48
		E		255	-	-	-	-
2015.5.6	159.4	F		-	-	-	251	273
		E		81	11	2	-	-

The light and dark grey backgrounds represent recovery group and flooding group samples, respectively.

Supplementary Table 2 Background and details of anthropogenic disturbance in two study areas.

Study area	Soil texture	Total grain output (ton year^{-1})	Poultry population	Agricultural fertilizer				Population density (people km^{-2})
				N-fertilizer (kg N year^{-1})	P-fertilizer (kg P year^{-1})	K-fertilizer (kg K year^{-1})	Compound Fertilizer (kg NPK year^{-1})	
AA	Silt clay	284	615	8754.3	2701.5	426.8	4025.5	485
NA	Silt clay	27	125	892.8	206.4	38.4	396.5	55

Notes: Data in each area were collected and gathered within 1 km on the basis of the statistical yearbook of Kaixian (Kaixian County Bureau of Statistics 2013 (In Chinese)) and social surveys conducted in 2013.

REFERENCE

Kaixian County Bureau of Statistics. *Statistical Yearbook of Kaixian County (2012)*. Chongqing, China Kaixian County Annals Editorial Department, 2013 (In Chinese).
