# Supplementary material for: Anthropogenic Pollution Intervenes the Recovery Processes of Soil Archaeal Community Composition and Diversity From Flooding
Source: Front Microbiol. 2019 Oct 2;10:2285. doi: 10.3389/fmicb.2019.02285 (PMC6783558; doi:10.3389/fmicb.2019.02285)
Supplement: Supplementary file 2 [file Data_Sheet_2.PDF]

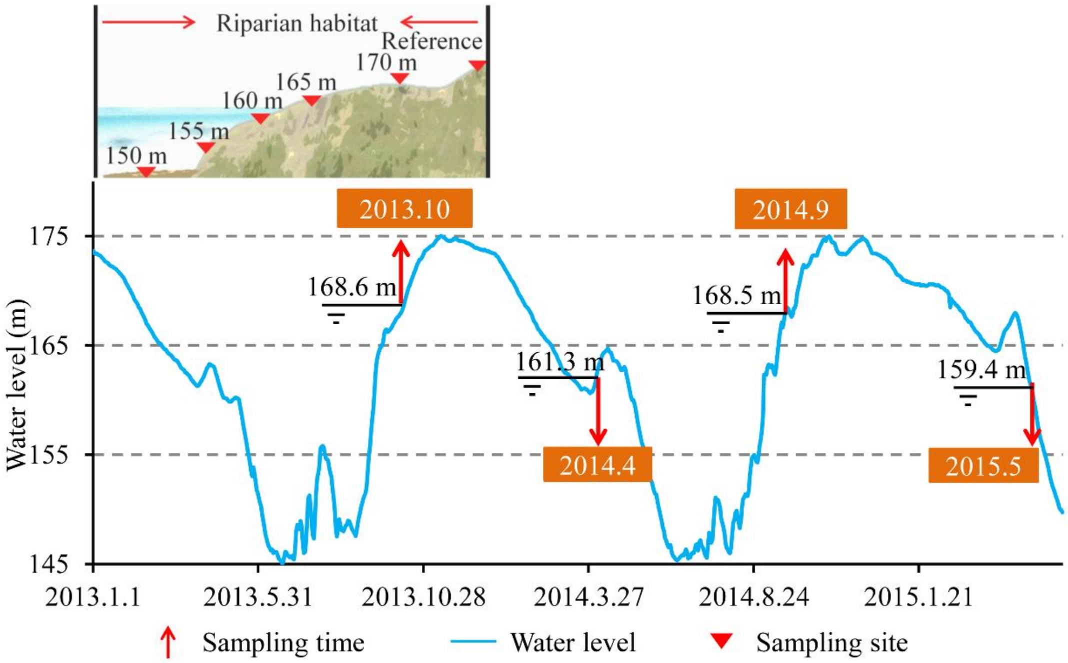

Supplementary Figure 1. Water level fluctuation and sites distribution at six elevations of the riparian habitat. Sampling dates are marked by arrows with corresponding water levels. Daily data of water level was collected from the official release of China Three Gorges Corporation (http://www.ctg.com.cn/).

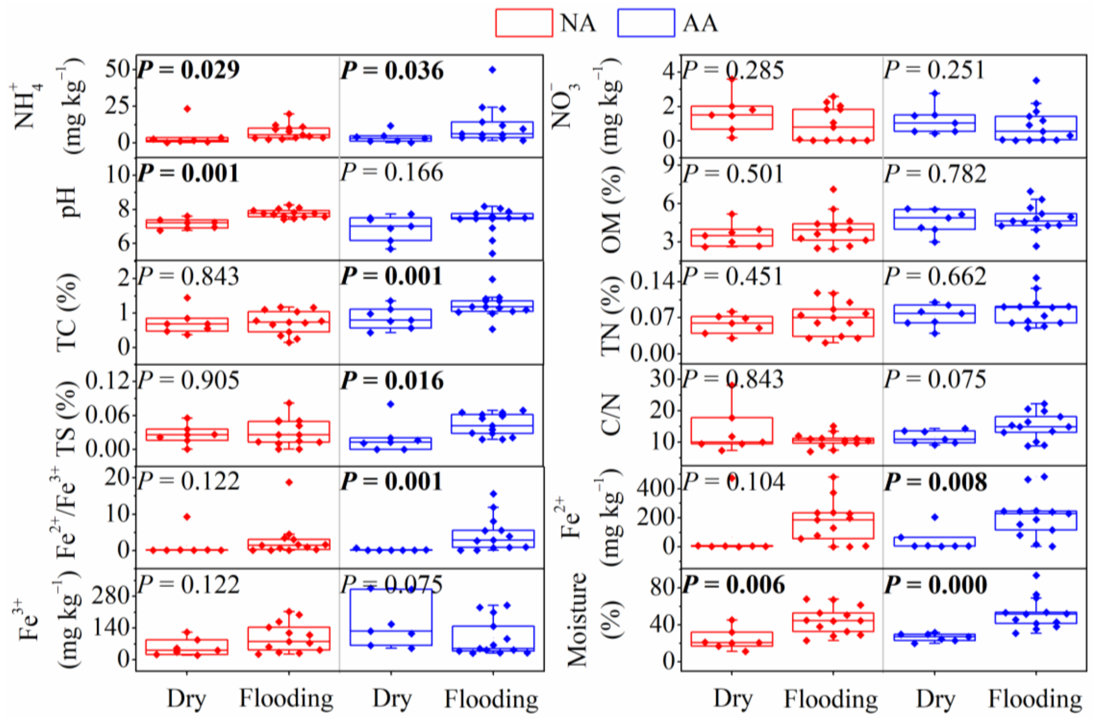

Supplementary Figure 2. Soil properties of NH_4^+, NO_3^- and pH in dry and flooding conditions. *P* values in bold indicate significant differences at 0.05 level based on Mann-Whitney *U*-test.

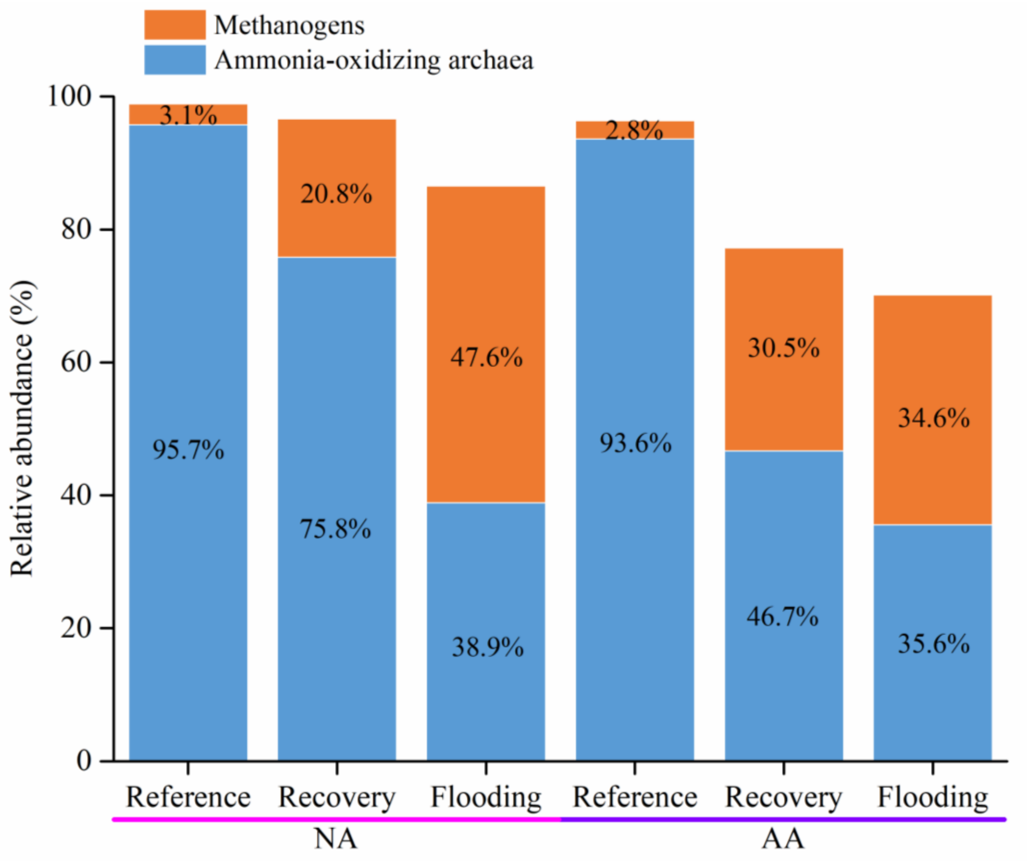

Supplementary Figure 3. Relative abundance of ammonia-oxidizing archaea and methanogens in the three sample groups in NA and AA.

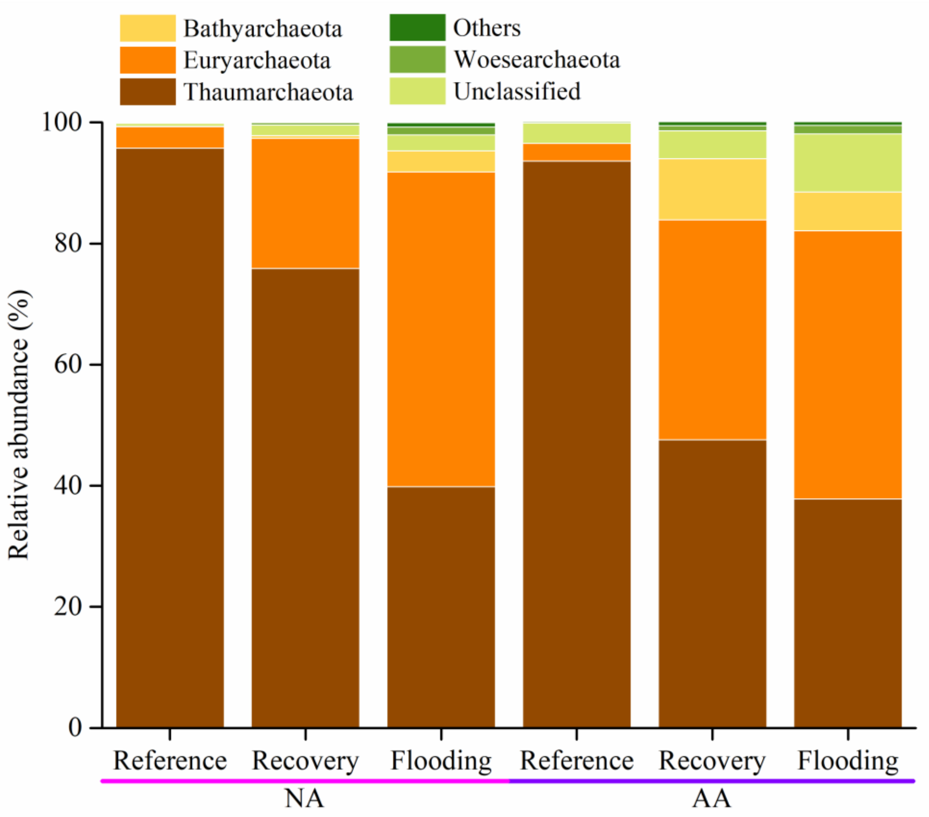

Supplementary Figure 4. Relative abundance of dominant phyla (relative abundance > 0.1%) in the three sample groups in NA and AA. The phylum with relative abundance < 0.1% in each group is grouped as "Others".
